# Domestic Medicine

**Published:** 1872-01

**Authors:** 


### Domestic Medicine.

### USEFUL RECIPES FOR THE HOUSEHOLD.

To REMOVE GREASE SPOTS.—Take equal parts of the spirits of camphor and ammonia. Mix them, saturate a cloth with the mixture, and rub upon the grease-spot. It will remove it immediately.

---

To PRESERVE EGGS.—Take of quick lime and salt, each, one pound; Saltpetre, three ounces; Water, one gallon. Boil for ten or fifteen minutes, and when cold, put in the eggs, small end downwards, using a vessel lined with lead, and placed in a cold, dry cellar. So says the *Year Book of Pharmacy*.

---

To MAKE HARD SOAP.—Place in an iron kettle, four pounds of unslacked lime, five pounds of soda, and three gallons of soft water; let it stand over night; in the morning pour off the water, and add to it three and a half pounds of grease; boil till thick, turn into a pan until cool, and then cut in bars.

---

To PREVENT PITTING IN SMALLPOX.—Make a sort of salve of pulverized charcoal and lard, by rubbing them together in a mortar, or in a deep dish. Smear it upon soft cloths, and apply to the face, neck and hands, as soon as the disease is recognized, and continue it until the fever has abated. This application allays the itching, prevents pitting, and seems to shorten the duration of the disease.

---

MUCILAGE OF GUM TRAGACANTH.—You can make an excellent mucilage that will never sour, and will always be ready for the various demands for it in every household:

Take Pulverized tragacanth, 1 drachm;
Glycerine, 6 drachms;
Water, enough to make 10 ounces.

Rub the tragacanth with the glycerine in a mortar, or on a dinner plate, then add the water.

---

FOR BALDNESS.—The following preparation will be found to be an excellent one for the cure of baldness, when the hair follicle has not been destroyed. Hair *cannot* be made to grow where the hair follicle is dead; but, in case where the hair is falling out, or when the follicle is obstructed by sebaceous matter, preventing the hair from coming through to the surface, the wash here recommended, will be found to be excellent:

Take
Bay rum, 1 quart;
Tincture of cantharides, 2 ounces;
Carbonate of potash,
Carbonate of ammonia, *of each*, 22 grs.

Dissolve the carbonate of potash and ammonia in a little water. Then mix with the other liquids.

Wet the scalp with the preparation, and brush with a stiff brush or crash towel. Then wash the scalp with warm water. Afterward, use simple bay rum as a dressing. Never use oils of any kind. The mixture should be applied once in three days.

---

FOR CHAPPED LIPS.—Now, that the cold weather with its bleak winds are upon us, our lips, faces and hands, are apt to become chapped. This can be prevented or cured by means of the following pomatum:

Take

| | | |
|---|---|---|
| Purified Lard, | 16 | parts; |
| Cacao oil, | 24 | " |
| Spermaceti, | 3 | " |
| Alcannæ, | 1 | " |

These substances are melted together, by gentle heat, then strained through a cloth and mixed with,

| | | |
|---|---|---|
| Oil of lemon, | 1-6 | part; |
| Oil of bergamot, | 1-6 | " |
| Oil of bitt'r almonds, | 1-15 | " |

after which pour the mass into a suitable dish to cool. It will be found a delightful preparation for chapped skin.

---

COLOGNE WATER.—You can make cologne water, for general use, much cheaper than you can *buy* it. The following formula will make a very superior article.

Take of
pure alcohol, 3 gallons;
Oil of neroli, 2 ounces;
" rosemary, 1 ounce;
" orange, $2\frac{1}{2}$ ounces;
" citron, $2\frac{1}{2}$ ounces;
" bergamot, 1 ounce.

Mix, and shake thoroughly; then allow it to stand for a few days perfectly quiet, before bottling. For a smaller quantity use the same ingredients proportionably reduced.

FOR PIN OR THREAD WORMS.—These troublesome little animals may be destroyed in the following manner:

Take of
- Senna,
- Rheubarb,
- Santonica,
- Tansy,
- Irish moss,
- Wormwood, each, one drachm. Add to this 7½ ounces, by weight, (avoird.), of water, with enough sugar to make a syrup. Allow it to stand in a bottle, and, without shaking it, pour from the top; and, to a child three years old, give a teaspoonful every morning for three days.

---

GERM THEORY—JAM PRESERVING.—The question of germ theory has a direct bearing upon domestic economy, when applied to jam preserving. After the cups or jars are filled with the jam or preserves, they are generally set one side to cool, before they are covered with the customary oiled paper. This affords the germs in the atmosphere an opportunity to decend upon the rich and fertile "soil," the result being a plentiful crop of mould. It must be known, that this mould is a vegetable growth, and when examined under the microscope, presents the appearance of a beautiful forest of trees, of various forms and sizes. Therefore, when it is desired to prevent this crop from appearing upon the surface of the jam, it is only necessary to cover it *before* it gets cool. In this manner you shut out the germs, all those that fell upon it while hot, being destroyed. Remember this ladies, when you engage in the jam and preserving business next Summer.

---

SUN FLOWER SEED.—The following valuable properties of this familiar plant are mentioned in the *American Journal of Pharmacy*:

"The great variety of valuable properties belonging to the sun-flower seed, have been much neglected. No plant produces such fine honey and wax, and when the flower is in blossom, bees abound in it. The produce will be according to the nature of the soil, and mode of cultivation; but the average has been found to be fifty bushels of seed to the acre, which will yield fifty gallons of oil. The oil is excellent, when refined, for table use, for burning in lamps, for soap making, and for painting—especially, for mixing green and blue paints. The marc, or refuse of the seed of the above quantity after the oil has been expressed, made into cakes, will produce 1500 lbs., and the stalks when burnt for alkali, will give 10 per cent. of potash. The green leaves of the sun-flower, when dried and burnt to powder, mixed with bran, makes excellent fodder for milch cows. It makes a beautiful soap, particularly softening to the hands and face, and is pleasant to shave with. The cake is superior to linseed, for fattening cattle. Sheep, pigs, pigeons, rabbits, poultry, of all sorts, etc., will fatten rapidly upon it, and prefer the seed to any other; it causes chickens to have much more glossy plumage, and to be plumper in the body. It also increases the quantity of eggs. The seed, shelled, makes when ground, very fine flour for bread, particularly, tea-cakes."

We know of no seed, possessing more valuable and versatile properties, than the sun-flower. Should think every farmer would raise a crop, even if it is *half* as good as represented.—ED. BISTOURY.

---

INTERNAL PILES.—In answer to several correspondents who have written us for formulæ, for the relief of this troublesome affection, we give below two recipes, which will generally cure hæmorrhoids, without resorting to a surgical operation.

Any intelligent druggist will prepare the medicine by showing him the formulæ:

Take
- Pulv. Senna,
- Bitartrate of Potass.,
- Pulv. Sulphur, *of each*, 2 oz.;
- Pulv. Zinziber, ½ oz.

Mix, and take a teaspoonful of the powder, in syrup, every morning, before breakfast.

Also, have prepared the following ointment:

Take
- Extract of belladonna,
- Sugar of lead, *of each*, 2 drachms,
- Tannin, ½ oz.,
- Simple cerate, a sufficient quantity to make a salve.

A small quantity of the ointment may be introduced within the anus, three times a day, first having bathed the parts thoroughly in cold water. A cold sitz bath every night, before retiring, will be found highly beneficial. The most obstinate and painful forms of hæmorrhoids can be entirely cured, in from one to five weeks, if this treatment is thoroughly persevered in.

